# A multicentre phase II trial of bryostatin-1 in patients with advanced renal cancer

**DOI:** 10.1038/sj.bjc.6601321

**Published:** 2003-10-14

**Authors:** S Madhusudan, A Protheroe, D Propper, C Han, P Corrie, H Earl, B Hancock, P Vasey, A Turner, F Balkwill, S Hoare, A L Harris

**Affiliations:** 1 Cancer Research UK Medical Oncology Unit, Churchill Hospital, Oxford, UK; 2 Department of Oncology, Addenbrooke's Hospital, Cambridge UK; 3 Department of Clinical Oncology, Weston Park Hospital, Sheffield, UK; 4 Beatson Oncology Centre, Western Infirmary, Glasgow, UK; 5 Drug Development Office, Cancer Research UK, 61 Lincoln's Inn Fields, London, UK; 6 Cancer Research UK Translational Oncology Laboratory, Barts & The London, Queen Mary's Medical School, Charterhouse Square, London UK

**Keywords:** kidney cancer, protein kinase C, bryostatin-1, phase II trial

## Abstract

Protein kinase C (PKC) has a critical role in several signal transduction pathways, and is involved in renal cancer pathogenesis. Bryostatin-1 modulates PKC activity and has antitumour effects in preclinical studies. We conducted a multicentre phase II clinical trial in patients with advanced renal cancer to determine the response rate, immunomodulatory activity and toxicity of bryostatin-1 given as a continuous 24 h infusion weekly for 3 out of 4 weeks at a dose of 25 *μ*g m^−2^. In all, 16 patients were recruited (11 males and five females). The median age was 59 years (range 44–68). Patients had been treated previously with nephrectomy (8) and/or interferon therapy (9) and/or hormone therapy (4) and/or radiotherapy (6). Eight, five and three patients had performance statuses of 0, 1 and 2, respectively. A total of 181 infusions were administered with a median of 12 infusions per patient (range 1–29). Disease response was evaluable in 13 patients. Three patients achieved stable disease lasting for 10.5, 8 and 5.5 months, respectively. No complete responses or partial responses were seen. Myalgia, fatigue, nausea, headache, vomiting, anorexia, anaemia and lymphopenia were the commonly reported side effects. Assessment of biological activity of bryostatin-1 was carried out using the whole–blood cytokine release assay in six patients, two of whom had a rise in IL-6 levels 24 h after initiating bryostatin-1 therapy compared to pretreatment values. However, the IL-6 level was found to be significantly lower at day 28 compared to the pretreatment level in all six patients analysed.

Renal cancer accounts for 2% of all cancers and the global incidence is increasing (Parkin *et al*, 1999; Mathew *et al*, 2002). Current modalities of therapy (including nephrectomy and/or immunotherapy) have only modestly improved the survival rates (Glaspy, 2002). Hence, there is a need to identify novel molecular targets and cancer therapies to improve patient outcomes.

Protein kinase C (PKC) is a calcium and lipid- activated serine-threonine protein kinase that has a central role in several signal transduction pathways and is involved in the pathogenesis of renal cancer (Pal *et al*, 1997; Fowler *et al*, 1998).

Bryostatin-1 is a highly oxygenated macrolide with a unique polyacetate backbone isolated from the marine bryozoan ‘Bigula neritina’. Bryostatin-1 causes isotype-specific PKC modulation and preclinical studies confirm its anticancer activity (Dale and Gescher, 1989; Kraft *et al*, 1989; Lilly *et al*, 1990; Schuchter *et al*, 1991; Stanwell *et al*, 1994). Bryostatin-1 also has significant immunomodulatory activity (Mohr *et al*, 1987; Tuttle *et al*, 1992; Philip *et al*, 1993).

Myalgia was the dose-limiting toxicity in several phase I human trials. The other commonly reported side effects were fever, flu-like symptoms, fatigue, anaemia, transient thrombocytopenia, phlebitis, headache, hypotension, bradycardia, flushing, dyspnoea, photophobia and eye pain (Prendiville *et al*, 1993; Grant *et al*, 1998; Weitman *et al*, 1999). Protein kinase C activity was significantly modulated during bryostatin-1 infusion in peripheral blood mononuclear cells (Varterasian *et al*, 1998). Previously, we reported a phase I trial of bryostatin-1, where increases in plasma IL-6 and TNF-*α* were seen within 24 h of therapy and partial responses were seen in two patients with melanoma (Philip *et al*, 1993). In another phase I trial of bryostatin-1 given as a 24 h intravenous infusion, weekly for 8 weeks, dose-limiting myalgia occurred at a dose of 25 *μ*g m^−2^. Four patients achieved favourable disease responses (two patients had partial responses and two had minor responses) (Jayson *et al*, 1995).

Based on the above biochemical, preclinical and clinical data, we initiated a multicentre phase II study to determine the response rate, immunomodulatory activity and toxicity of bryostatin-1 given as continuous 24 h intravenous infusion weekly for 3 out of 4 weeks at a dose of 25 *μ*g m^−2^ in renal cancer.

## MATERIALS AND METHODS

### Patient selection:

This multicentre phase II trial was conducted in compliance with the requirements of the declaration adopted by the world assemblies held at Helsinki, Tokyo, Venice and Hong Kong (1989). Each participating centre was required to obtain local research ethic committee approval prior to initiating the trial. All patients provided written informed consent. Patients were eligible to participate in the study if they had advanced, histologically confirmed renal cell carcinoma with bidimensionally measurable lesions and documented disease progression within 2 months prior to entry. A performance status of 0,1 or 2 and an expected life expectancy of greater than 3 months was necessary. Patients had to have normal full blood counts (granulocyte count>1.5 × 10^9^ l^−1^, total platelet count>100 × 10^9^ l^−1^), normal renal function (serum creatinine <150*μ*M) and acceptable liver function tests (serum bilirubin <20*μ*M, transaminases < 2.5 × normal). All patients were required to have a central venous catheter inserted before initiating therapy.

### Treatment plan

Bryostatin-1 was supplied by the (National Cancer Institute, NCI USA) as a two-part formulation. A 6 ml vial containing 0.1 mg bryostatin-1 and 5 mg povidone USP (as a bulking agent) lyophilised from 40% *t*-butanol and a second vial of sterile PET diluent (60% polyethylene glycol 400, 30% ethanol and 10% Tween 80). The lyophilised powder was reconstituted in 1 ml of the PET diluent and further diluted in 50 ml of 0.9% sodium chloride, USP. The resulting solution containing 2 *μ*g ml^−1^ of bryostatin-1 was transferred to a 50 ml non-PVC syringe. Brystatin-1 was administered at a dose of 25 *μ*g m^−2^ (concentration of 2 *μ*g ml^−1^) as a 24 h infusion (ambulatory pump) via central line weekly for 3 out of 4 weeks. As bryostatin-1 is adsorbed onto plastic surfaces, a ‘non- poly vinyl chloride (PVC) Gish skin tunnelled central venous catheter’ was inserted in all patients. Each 4-week block comprised one cycle of treatment.

### Evaluation protocol at baseline and during therapy

All participating patients were carefully monitored during therapy. History, physical examination and performance status assessments were performed at baseline, prior to each cycle of therapy and at the end of treatment. Biochemical evaluation (serum Na, K, glucose, urea, creatinine, alkaline phosphatase, total bilirubin, AST and GGT) and haematological evaluation (Hb, white cell count and platelet count) were performed at baseline, prior to each infusion of therapy and at the end of treatment. Radiological evaluation (CT scan, X-ray) was performed at baseline, after two cycles of treatment and 8 weekly thereafter until disease progression. For the assessment of biological responses, blood samples were collected during the first month of therapy at set time points (pretreatment, 24 h, days 15 and 28).

Patients were evaluated for radiological disease response after two cycles of therapy. Complete response (CR) was defined as disappearance of all clinical evidence of active tumour for a minimum of 4 weeks and normalisation of tumour markers and tumour-related biochemical abnormalities. Partial response (PR) was defined as 50% or greater decrease in the sum of the diameters of measured lesions. Stable disease (SD) was defined as <50% decrease or <25% increase in the sum of the longest perpendicular lesion diameter. An increase of >25% in the size of any measured lesion or appearance of new lesions was defined as progressive disease (PD). Patients who achieved SD or showed regressions after 2 months of therapy were planned to receive further courses of bryostatin-1 with disease response assessments done at 8-weekly intervals. Therapy was terminated at disease progression, with the occurrence of a serious adverse event or at patient's request.

Toxicity evaluation was performed using National Cancer Institute's common toxicity criteria (NCI-CTC Version 2.0). Toxicity was categorised as unlikely, possibly, probably or almost certainly related to bryostatin-1. For patients who experienced > grade 2 myalgia and/ or headache (lasting for more than 7 days) due to bryostatin-1, treatment delays for up to 1 week was allowed and therapy restarted if the above symptoms settled. However, for other grade 3–4 toxicity possibly, probably or definitely related to bryostatin-1, treatment was terminated. No dose modification was allowed in this study.

### Laboratory procedures

TNF-*α* mediates the production of IL-6 in the whole blood when stimulated by phytohaemagglutinin (PHA) *in vitro*. Therefore, whole-blood samples were collected from six patients at four different time points during a 28-day treatment cycle (pretreatment, 24 h, days 15 and 28). Blood samples were collected into tubes containing preservative-free heparin (30 *μ* ml^−1^ blood, Leo Laboratories Ltd, Princes Risborough, UK) and PHA (2 *μ*g ml^−1^ blood, HA 16/17, Murex Biotech Ltd, Dartford, UK), or heparin alone as controls. All samples were collected and processed under sterile and pyrogen-free conditions. Whole–blood samples were incubated at 37°C in a 5% CO_2_-saturated humidified incubator. At 24 h post *in vitro* stimulation with PHA, the blood samples were cool spun, the supernatant separated, flash frozen and stored at −20°C. Interleukin-6 (IL-6) was measured using the ELISA kit D6050 (R & D Systems, Abingdon, UK) and TNF-*α* was measured using DTA50 (R & D Systems, Abingdon, UK).

### Statistical analysis

Wilcoxon matched-pairs signed-ranks test was used to analyse changes in TNF-*α* and IL-6 levels with therapy (pretreatment *vs*. 24 h sample and pretreatment *vs*. day 28 sample).

## RESULTS

### Patient characteristics

Baseline data for the patients are summarised in Table 1

**Table 1:** Baseline data

*Total Number of patients*	16
Males	11
Females	5
	
*Median age*	59 years (range 44–68)
Previous therapy (number of patients)	
Nephrectomy	8
Interferon	9
Hormone therapy (megesterol/medroxyprogesterone)	4
Radiotherapy	6
	
*Time from diagnosis to systemic therapy (number of patients)*	
0–24 months	13
⩾24 months	3
	
*Baseline WHO performance status (number of patients)*	
Zero	8
One	5
Two	3
	
Total number of bryostatin-1 infusions	181 (median 12 (range 1–29))

. Eight patients had synchronous metastatic disease (i.e. primary disease and metastasis) at presentation. Biopsy was performed for histopathological diagnosis in these patients. They did not receive adjunctive nephrectomy. The majority of patients (13 patients) had more than one site of metastatic disease. Three patients had lung metastasis only at the time of entry into the trial. A total of 181 infusions were administered with a median of 12 infusions per patient (range 1–29). In all, 59 completed cycles were administered; 10 patients received more than 2 and six patients received 2 or less cycles of therapy.

### Disease response

A total of 13 patients were available for disease response evaluation (three patients were not evaluable, one patient had one cycle of therapy and refused further participation in the study, one patient had two cycles but refused evaluation and one patient received less than one cycle and died due to an unrelated cause). In 10 patients, radiological assessment performed after the second cycle of therapy showed no disease progression. Therefore, they continued to receive bryostatin-1 beyond cycle two. Three patients achieved stable disease (SD) lasting for 10.5, 8 and 5.5 months, respectively. Patient one was treated with nephrectomy at initial presentation. He relapsed with lung metastasis 15 months later and received interferon therapy. However, he progressed within 3 months of interferon treatment. He achieved stable disease for 10.5 months while on bryostatin-1 therapy. Patient two, who presented with synchronous metastatic disease, received interferon as first-line treatment. Although he progressed within 4 months of interferon therapy, he achieved stable disease lasting for 8 months on bryostatin-1. He was later withdrawn from the trial because he developed grade 4 haematuria possibly related to bryostatin-1. No complete responses or partial responses were seen. In total, 10 patients had progressive disease within 4 months of initiating bryostatin-1 therapy.

### Toxicity data

Bryostatin-1 therapy was reasonably well tolerated. Myalgia (grade 3–2 pts, grade 2–2 pts, grade 1–4 pts), fatigue (grade 2–3 pts, grade 1–4 pts), nausea (grade 2–3 pts, grade 1–5 pts), headache (grade 2–3 pts), vomiting (grade 1–3 pts), anorexia (grade 1–3 pts), anaemia (grade 2–6 pts, grade 1–1 pts) and lymphopenia (grade 2–6 pts, grade 1–4 pts) were the commonly reported side effects probably, possibly or almost certainly related to bryostatin-1. There were no treatment-related deaths. One patient died 4 days after the first bryostatin-1 infusion. He presented acutely unwell with worsening renal functions. He suffered cardiac arrest and died. A detailed post-mortem examination reported perforated duodenal ulcer as the cause of death. This was not considered to be related to bryostatin-1. Dyskinetic tongue movements possibly related to bryostatin-1 were seen in another patient. Spontaneous grade 4 haematuria was seen in one patient. This was considered to be due to bryostatin-1 and he was withdrawn from the trial. Another patient developed grade 4 skin rash manifesting as scaly and ulcerating lesion on the legs possibly related to bryostatin-1 (withdrawn from trial later at the patient's request). Other grade 1 or 2 side effects reported were fever (one patient), constipation (one patient), arthralgia (one patient), abdominal pain (three patients), conjunctivitis (one patient), blurred vision (one patient), insomnia (two patients), anxiety (one patient), palpitations (one patient) and phlebitis (one patient). Only one patient had catheter-related infection and inflammation (unrelated to byrostatin-1). Grade 1 and 2 biochemical abnormalities reported possibly were hypocalcaemia (five patients), hypoglycaemia (three patients), hyponatraemia (three patients), and hyperkalaemia (two patients).

### Biological activity

**Figure 1:**
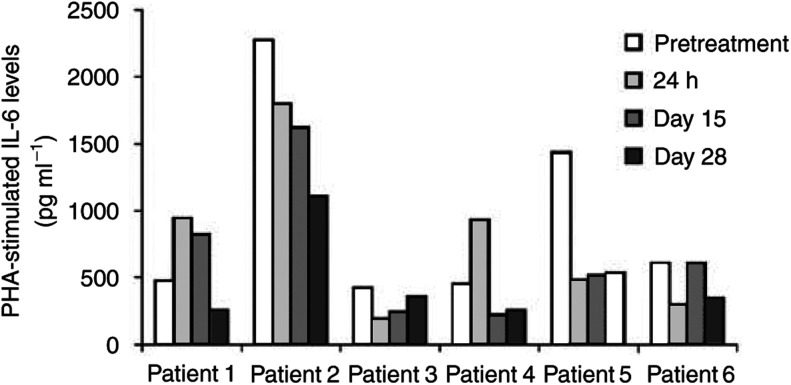
Illustrates IL-6 release in the whole-blood cytokine assay. Samples were taken at set time points during therapy (pretreatment, 24 h, days 15 and 28). IL-6 was measured by ELISA. A rise in IL-6 levels was seen in two patients (patients 1 and 4) after 24 h of bryostatin-1. After 28 days therapy, the production of IL-6 was reduced in all the six patients (*P*=0.03).

Assessment of biological activity of bryostatin-1 was performed in six patients using the whole-blood cytokine release assay (Thavasu *et al*, 1999). Briefly, whole blood from patients was stimulated with PHA and release of cytokines IL-6 and TNF-*α* measured after 24 h incubation at 37°C. The ability of bryostatin-1 therapy to alter this response was assessed in sequential samples over a 28-day time period (Figure 1

). IL-6 production was significantly reduced in all the six patients at day 28 compared to pretreatment values (*P*=0.03). The mean pretreatment IL-6 level was 943 pg ml^−1^ (range 420–2271, standard deviation 755). The mean IL-6 level at day 28 was 476 pg ml^−1^ (range 253–1107, standard deviation 325). There was no correlation between IL-6 level and disease response. All the six patients who had reduced IL-6 response at day 28 progressed within 3 months of therapy. In three patients who had disease stabilization, IL-6 level assessments were not performed. Bryostatin-1 treatment did not affect the levels of TNF-*α* produced in the whole-blood cytokine assay.

## CONCLUSIONS

A total of 16 patients with metastatic renal cancer were recruited into this multicentre phase II clinical trial. Three patients achieved stabilisation of disease lasting for 10.5, 8 and 5.5 months, respectively. No complete or partial responses were observed. Bryostatin-1 was reasonably well tolerated and most of the side effects were as expected from previous phase I studies. One patient died 4 days after the first bryostatin-1 infusion. Perforated duodenal ulcer was the cause of death in this patient and was not considered to be due to bryostatin-1. Sudden unexplained death was reported in a patient in another phase II trial of bryostatin-1 (Pagliaro *et al*, 2000). Dyskinetic tongue movements seen in one patient have not been reported before. Anaemia and lymphopenia were of no obvious clinical consequence. The mechanisms of biochemical abnormalities (hyponatraemia, hypocalcaemia, hypoglycaemia and hyperkalaemia) due to bryostatin-1 are unknown. Hyponatraemia was seen in five patients with head and neck cancer in another phase II trial of bryostatin-1 (Brockstein *et al*, 2001). We investigated the role of IL-6 and TNF-*α* as potential markers of biological effect of bryostatin-1. While the previously reported studies investigated IL-6 and TNF-*α* levels within 24 h after the initiation of bryostatin-1 therapy (Philip *et al*, 1993; Jayson *et al*, 1995), we extended the time points to up to day 28 to assess the long-term biological effect of bryostatin-1. Two patients had a rise in IL-6 levels after 24 h of therapy. However, all the six patients tested showed reduction in IL-6 levels at day 28, which was statistically significant. This probably correlates with the *in vitro* effect of bryostatin-1, where initial activation of PKC is followed by its rapid downregulation. Reduction of IL-6 after repeated bryostatin-1 infusions may represent the biological consequence of this effect on the signal transduction pathway. Interestingly, recent studies confirm the role of IL-6 in promoting angiogenesis and cancer progression (Salgado *et al*, 1999,^2002^; Benoy *et al*, 2002).

Phase II trials of bryostatin-1 have been reported in many tumour types and several patients have achieved disease stabilisations in these studies (Propper *et al*, 1998; Gonzalez *et al*, 1999; Bedikian *et al*, 2001; Brockstein *et al*, 2001). These trials have used different doses and schedules of bryostatin-1. A previously published phase II trial in kidney cancer patients used bryostatin-1 at a dose of 25 *μ*g m^−2^ administered as a 30 min intravenous infusion on days 1, 8 and 15 of a 28-day cycle. Two patients had objective responses but methodological problems made disease response assessment difficult (Pagliaro *et al*, 2000). Bryostatin-1 used at a dose of 35–40 *μ*g m^−2^ given intravenously over 1 h on days 1, 8 and 15 of a 4-weekly cycle produced prolonged stable disease or partial response in 25% of patients with renal cancer in a recently reported phase II trial (Haas *et al*, 2003). Experimental data suggest that bryostatin-1 may have a synergistic anticancer effect with chemotherapy (Mohammad *et al*, 2000). In fact, early- phase human trials of bryostatin-1 in combination with chemotherapy (vincristine, paclitaxel and cisplatin) seem to suggest that this approach is feasible and safe (Kaubisch *et al*, 1999; Rosenthal *et al*, 1999; Varterasian *et al*, 2000). Newer bryostatin analogues have also been synthesised recently and their role as anticancer agents needs investigation (Wender *et al*, 1999). We conclude that bryostatin-1 monotherapy does not have a major impact on renal cancer metastasis. Future trials in combination with immunotherapy or chemotherapy or the use of newer more potent bryostatins may be more promising.
